# The Synthesis and Antitumor Activity of Twelve Galloyl Glucosides

**DOI:** 10.3390/molecules20022034

**Published:** 2015-01-27

**Authors:** Chang-Wei Li, Hua-Jin Dong, Cheng-Bin Cui

**Affiliations:** State Key Laboratory of Toxicology and Medical Countermeasures, Beijing Institute of Pharmacology and Toxicology, Beijing 100850, China; E-Mails: sdrlcw@126.com (C.-W.L.); huajind@hotmail.com (H.-J.D.)

**Keywords:** galloyl glucoside, synthesis, antitumor activity

## Abstract

Twelve galloyl glucosides **1–12**, showing diverse substitution patterns with two or three galloyl groups, were synthesized using commercially available, low-cost d-glucose and gallic acid as starting materials. Among them, three compounds, methyl 3,6-di-*O*-galloyl-*α*-d-glucopyranoside (**9**), ethyl 2,3-di-*O*-galloyl-*α*-d-glucopyranoside (**11**) and ethyl 2,3-di-*O*-galloyl-*β*-d-glucopyranoside (**12**), are new compounds and other six, 1,6-di-*O*-galloyl-*β*-d-glucopyranose (**1**), 1,4,6-tri-*O*-galloyl-*β*-d-glucopyranose (**2**), 1,2-di-*O*-galloyl-*β*-d-glucopyranose (**3**), 1,3-di-*O*-galloyl-*β*-d-glucopyranose (**4**), 1,2,3-tri-*O*-galloyl-*α*-d-glucopyranose (**6**) and methyl 3,4,6-tri-*O*-galloyl-*α*-d-glucopyranoside (**10**), were synthesized for the first time in the present study. In *in vitro* MTT assay, **1–12** inhibited human cancer K562, HL-60 and HeLa cells with inhibition rates ranging from 64.2% to 92.9% at 100 μg/mL, and their IC_50_ values were determined to be varied in 17.2–124.7 μM on the tested three human cancer cell lines. In addition, compounds **1–12** inhibited murine sarcoma S180 cells with inhibition rates ranging from 38.7% to 52.8% at 100 μg/mL in the *in vitro* MTT assay, and *in vivo* antitumor activity of **1** and **2** was also detected in murine sarcoma S180 tumor-bearing Kunming mice using taxol as positive control.

## 1. Introduction

Galloyl glucosides, a kind of plant polyphenolics, display various important, diverse biological and pharmacological activities, such as virustatic [1], anti-oxidant and free radical scavenging [2,3,4], interaction with protein [5] and enzyme inhibitory [6,7], anti-inflammatory [8], neuronal cell protecting [9], transient global ischemia/reperfusion-induced brain injury protecting [10], keratinocyte proliferation and mitochondrial activity stimulating [11], anti-diabetic [12], cisplatin-induced nephrotoxicity protecting [13] and antitumor [14,15,16,17,18] activities, and so on. Generally, galloyl glucosides are widespread in various plants, but the low content of their free form in plants and the high polarity, coupled with their easily oxidized chemical features, have made it difficult to isolate them in large amounts. The most highly galloylated one, penta-*O*-galloyl-*β*-d-glucopyranose (PGG), can be obtained in large amounts by acidic methanolysis of tannic acid from plants [6,16,19,20] or chemical synthesis [20,21]. Major biological studies on galloyl glucosides were also developed using PGG [5,6,7,8,9,10,11,12,13,14,15,16,17,18,19,20,21,22,23,24]. Boththe *in vitro* [13,14,15,16,17,20,21,22,23,24] and *in vivo* [14,20,21,24] antitumor activities of PGG had been verified and possible mechanisms had also been explored to some extent [20,21]. For instance, the following events, but not limited to, were reported to be probably responsible: the inhibition of DNA polymerases [16], inhibition of fatty acid synthase (FS) in FS-highly expressing glioma cancer U251 cells [14], induction of apoptosis by activating caspase-3 in HL-60 cells [22], inhibition of VEGF binding to VEGF receptor to exert anti-angiogenic effect [23], and activation of p53 tumor suppressor pathway and inhibition of STAT3 oncogenic signaling [24], and so on. In contrast, biological properties of other galloyl glucosides were not yet sufficiently investigated, probably because of the difficulty in obtaining sufficient amounts for study. Although the synthesis of some galloyl glucosides has been reported [25,26,27,28,29], in view of their diverse and important biological activities, it is still worthwhile to synthesize other galloyl glucosides with less galloyl groups than PGG to further investigate their biological properties.

**Figure 1:**
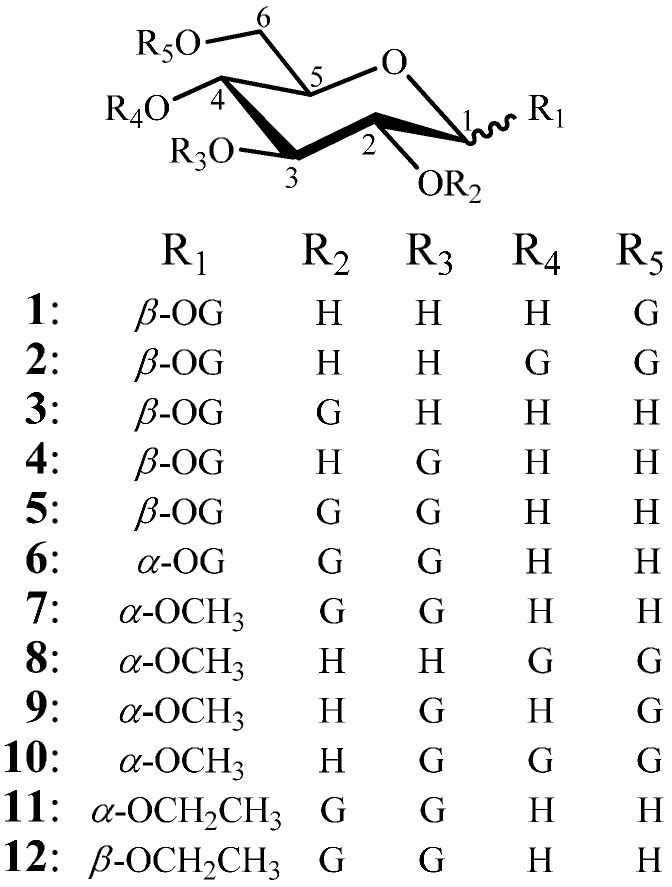
Chemical structures of twelve galloyl glucosides **1–12**.

In the course of our studies on bioactive natural products [30,31,32,33,34,35,36,37,38,39,40,41,42,43,44], we have reported the isolation of some phenolic compounds with antitumor, anti-hypoxia, and anti-bacterial activities, including several galloyl glucosides with one to four galloyl groups, from a medicinal plant, *Choerospondias axillaries*, which is used as a traditional herbal medicine in China [41,42,43,44]. Previously, we also reported the inhibitory effect of the galloyl glucosides with one to four galloyl groups on human cancer K562 cells, pointing out the stronger activity of the galloyl glucosides with two and three galloyl groups than the others [44]. As an extension of that work, and in view of our previous assay results [44], we recently synthesized twelve galloyl glucosides **1–12** with two or three galloyl groups, (in Figure 1), and evaluated their antitumor activity by *in vitro* MTT assay and/or *in vivo* test on tumor-bearing mice. Three of them, **9**, **11** and **12**, are new compounds and other six, **1–4**, **6** and **10**, were synthesized for the first time in the present study. The synthesis and antitumor activity of **1–12** were reported in detail in this paper.

## 2. Results and Discussion

### 2.1. Synthesis of **1–12**

#### 2.1.1. Synthesis of Esterification Reagent **13** and Intermediates **14–17**

**Scheme 1:**
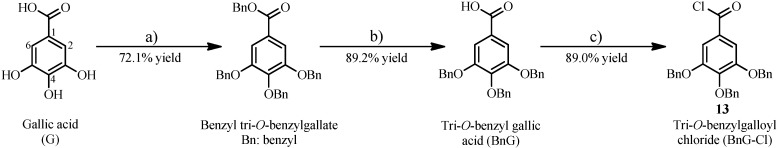
Preparation of tri-*O*-benzylgalloyl chloride (**13**).

Tri-*O*-benzylgalloyl chloride (BnG-Cl) (**13** in Scheme 1) was prepared in 57.2% overall yield using gallic acid as starting material by benzylation with benzyl chloride (Bn-Cl), hydrolysis, and then chlorination, as shown in Scheme 1.

*Reagents and conditions*: (a) K_2_CO_3_, PhCH_2_Cl, DMSO, 140 °C, 8 h; (b) NaOH, H_2_O/MeOH, 90 °C, 5 h; (c) SOCl_2_, 90 °C, 9 h.

**Scheme 2:**
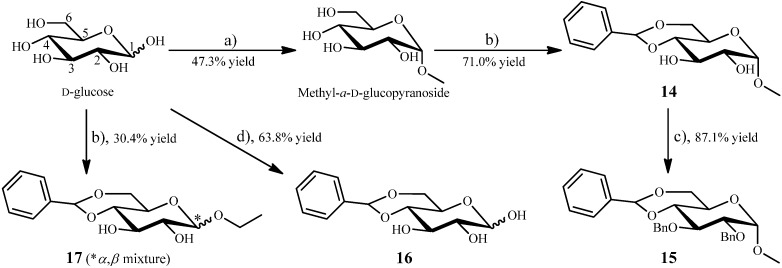
Preparation of **14–17** from d-glucose.

The intermediates **14–17** with a d-glucopyranose core were prepared from d-glucose, as shown in Scheme 2, and were used as starting materials for further synthesis of **1–12**. Acid-catalyzed methylation of d-glucose by methanol (MeOH) and acetyl chloride (Ac-Cl) gave methyl *α*-d-glucopyranoside in 47.3% yield, which, in turn, afforded methyl 4,6-*O*-benzylidene-*α*-d-glucopyranoside (**14**) in 71.0% yield by reaction with benzaldehyde (PhCHO) using *p*-toluenesulfonic acid (*p*TSA) as catalyst and triethyl orthoformate as dehydrator in tetrahydrofurane (THF) at 85 °C for 16 h (Scheme 2). Compound **14** was reacted with benzyl bromide (PhCH_2_Br) in the presence of sodium hydride (NaH) in *N*,*N*-dimethylformide (DMF) at room temperature for 4 h (Scheme 2). Then, the reaction mixture was suspended in water and extracted with chloroform (CHCl_3_) to obtain a CHCl_3_ solution. The CHCl_3_ solution was washed by water and then dried with anhydrous magnesium sulfate (MgSO_4_) to remove remaining sodium hydroxide (NaOH, from NaH) in CHCl_3_. The dried CHCl_3_ solution was then evaporated, at lower temperature at first to remove CHCl_3_ and further at 90 °C to remove remaining DMF, to obtain a CHCl_3_ extract. The whole CHCl_3_ extract was separated by column chromatography, followed by recrystallization, to give methyl 2,3-di-*O*-benzyl-4,6-*O*-benzylidene-*α*-d-glucopyranoside (**15**) in 87.1% yield.

*Reagents and conditions*: (a) MeOH, Ac-Cl, refluxing, 72 h; (b) PhCHO, triethyl orthoformate, *p*-toluenesulfonic acid monohydrate, THF, 85 °C, 16 h; (c) PhCH_2_Br, NaH, DMF, at room temperature, 4 h; (d) PhCHO, *p*-toluenesulfonic acid monohydrate, DMF, 85 °C, 16 h. * The intermediate **17** was obtained as a mixture of *α,β*-anomers.

On the other hand, direct treatment of d-glucose with benzaldehyde and *p*TSA catalyst in DMF at 85 °C for 16 h gave 4,6-*O*-benzylidene-d-glucopyranose (**16**) in 63.8% yield (Scheme 2). But when the reaction was performed in the presence of triethyl orthoformate in THF at 85 °C for 16 h, it produced **17** in 30.4% yield as a mixture of *α,β*-anomers, as shown in Scheme 2, which was separated by HPLC to give ethyl 4,6-*O*-benzylidene-*α*-d-glucopyranoside (**17a**) and ethyl 4,6-*O*-benzylidene-*β*-d-glucopyranoside (**17b**). In the case of this reaction, in addition to 4,6-*O*-benzylidene ring formation, triethyl orthoformate underwent hydrolysis to produce ethanol (EtOH), which, in turn, further reacted with reactive 1-OH in glucose at the acidic condition to produce **17**.

#### 2.1.2. Synthesis of **1** and **2** from the Intermediate **15**

**Scheme 3:**
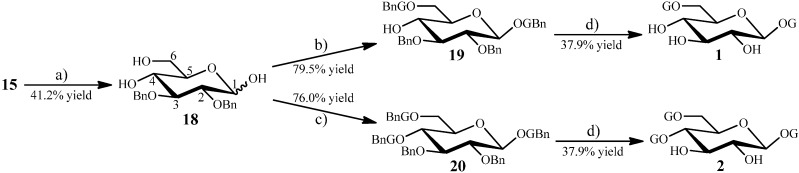
Synthesis of di-*O*- and tri-*O*-galloyl-d-glucosides **1** and **2**, from **15**.

The aimed two galloyl glucosides, 1,6-di-*O*-galloyl-*β*-d-glucopyranose (**1**) and 1,4,6-tri-*O*-galloyl-*β*-d-glucopyranose (**2**), were synthesized using **15** (in Scheme 2) as starting material, as shown in Scheme 3. Acid-catalyzed hydrolysis of **15** (in Scheme 2) by 2 N HCl at 80 °C for 16 h gave 2,3-di-*O*-benzyl-d-glucopyranose (**18**, in Scheme 3) in 41.2% yield. As shown in Scheme 3, reaction of **18** with BnG-Cl (**13**, in Scheme 1) in 1:2.7 molar ratio in anhydrous pyridine at 60 °C for 18 h afforded 1,6-*O*-diesterified product **19** (79.5% yield), while reaction of **18** with BnG-Cl in 1:7.3 molar ratio also in anhydrous pyridine at 60 °C for 18 h gave 1,4,6-*O*-triesterified product **20** (76.0% yield). Both **19** and **20** were purified by column chromatography, and hydrogenation of **19** and **20** over 10% Pd-C in THF containing approximate 25% of aqueous 95% EtOH under 10 atmospheric pressure at 40 °C for 12 h produced **1** and **2** both in 37.9% yields, respectively (Scheme 3).

*Reagents and conditions*: (a) 2 N HCl, 80 °C, 16 h; (b) BnG-Cl, 3 g of **18** in 30 mL anhydrous pyridine, 80 °C, 18 h; (c) BnG-Cl, 4.5 g of **18** in 170 mL anhydrous pyridine, 80 °C, 18 h; (d) H_2_, 10% Pa-C, in THF containing approximate 25% of aqueous 95% EtOH, 40 °C, 10 atm, 12 h.

#### 2.1.3. Synthesis of **3–6** from the Intermediate **16**

**Scheme 4:**
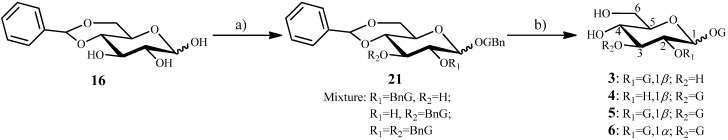
Synthesis of di-*O*- and tri-*O*-galloyl-d-glucosides, **3–4** and **5–6**, from **16** (in total 4.1%, 4.1%, 2.1% and 1.5% yields of **3**, **4**, **5** and **6** from **16**, respectively).

The target di- and tri-*O*-galloyl-d-glucosides, **3–4** and **5–6**, were synthesized using **16** (Scheme 2) as starting materials. Reaction of **16** with BnG-Cl (**13**, in Scheme 1) in 1:2.5 molar ratio in anhydrous pyridine at 60 °C for 48 h gave **21** as a mixture of 1,2-di-, 1,3-di- and 1,2,3-tri-*O*-esterified products, as shown in Scheme 4. Direct hydrogenation of the mixture **21** without separation over 10% Pd-C in THF containing 6.7% of aqueous 95% EtOH under 10 atmospheric pressure at 40 °C for 12 h, followed by chromatographic and HPLC separation, gave pure **3–6** in total 4.1% (**3**), 4.1% (**4**), 2.1% (**5**) and 1.5% (**6**) yields from **16**, respectively.

*Reagents and conditions*: (a) BnG-Cl, in anhydrous pyridine, 60 °C, 48 h; (b) H_2_, 10% Pa-C, in THF containing 6.7% of aqueous 95% EtOH, 40 °C, 10 atm, 12 h.

#### 2.1.4. Synthesis of **7** from the Intermediate **14**

**Scheme 5:**
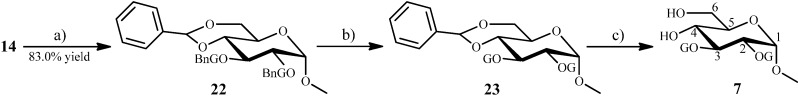
Synthesis of methyl 2,3-di-*O*-galloyl-*α*-d-glucopyranoside **7** from **14** (in total 64.5% yield of **7** from **22**).

The methyl 2,3-di-*O*-galloyl-*α*-d-glucopyranoside (**7**) could be synthesized from **14** (in Scheme 2), as shown in Scheme 5. Reaction of **14** (in Scheme 2) with BnG-Cl (**13**, in Scheme 1) in 1:4.1 molar ratio in anhydrous pyridine at 60 °C for 48 h gave the 2,3-di-*O*-esterified product **22** (83.0% yield). Hydrogenation of **22** on 10% Pd-C in THF containing approximate 13% of aqueous 95% EtOH under 10 atmospheric pressure at 40 °C for 12 h could reduce 2,3-di-*O*-GBn to 2,3-di-*O*-G, but could not remove protecting 4,6-benzylidene group in **22** to afford a partially deprotected product **23** (Scheme 5). Further acid-catalyzed hydrolysis of **23** by 1 N HCl at 55 °C for 6 h, followed by chromatographic separation, afforded **7** in total 64.5% yield from **22**.

*Reagents and conditions*: (a) BnG-Cl, in anhydrous pyridine, 60 °C, 48 h; (b) H_2_, 10% Pa-C, in THF containing approximate 13% of aqueous 95% EtOH, 40 °C, 10 atm, 12 h; (c) 1 N HCl, 55 °C, 6 h.

#### 2.1.5. Synthesis of **8** from the Intermediate **15**

**Scheme 6:**
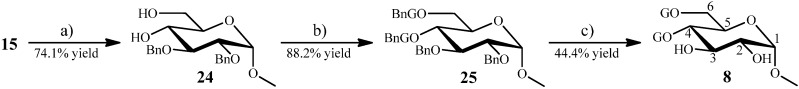
Synthesis of methyl 4,6-di-*O*-galloyl-*α*-d-glucopyranoside (**8**) from **15**.

The aimed methyl 4,6-di-*O*-galloyl-*α*-d-glucopyranoside (**8**) was synthesized from **15** (in Scheme 2) as shown in Scheme 6. Hydrolysis of 15 in 50% aqueous acetic acid (AcOH) at 55 °C for 24 h gave methyl 2,3-di-*O*-benzyl-*α*-d-glucopyranoside (**24**) in 74.1% yield (Scheme 6). Reaction of **24** with BnG-Cl (**13**, in Scheme 1) in 1:3.4 molar ratio in anhydrous pyridine at 60 °C for 48 h afforded 4,6-di-*O*-esterified product **25** in 88.2% yield, and hydrogenation of **25** on 10% Pd-C in THF containing 13% of aqueous 95% EtOH under 10 atmospheric pressure at 40 °C for 12 h produced **8** in 44.4% yield (Scheme 6).

*Reagents and conditions*: (a) 50% AcOH, 55 °C, 24 h; (b) BnG-Cl, in anhydrous pyridine, 60 °C, 48 h; (c) H_2_, 10% Pa-C, in THF containing approximately 13% of aqueous 95% EtOH, 40 °C, 10 atm, 12 h.

#### 2.1.6. Synthesis of **8–10** from the Intermediate **14**

**Scheme 7:**
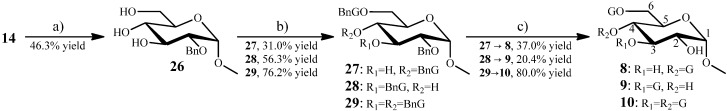
Synthesis of **8–10** from **14**.

Two target compounds, methyl 3,6-di-*O*-galloyl-*α*-d-glucopyranoside (**9**) and methyl 3,4,6-tri-*O*-galloyl-*α*-d-glucopyranoside (**10**) could be synthesized from **14** (in Scheme 2), as shown in Scheme 7. Meanwhile, methyl 4,6-di-*O*-galloyl-*α*-d-glucopyranoside (**8**) could be also synthesized from **14** with the synthesis of **9**. First, the key intermediate **26** (Scheme 7) was synthesized by reaction of **14** and benzyl bromide. Compound **14** was reacted with benzyl bromide in presence of NaH in DMF at room temperature for 6 h. Then, the reaction mixture was suspended in water and extracted with CHCl_3_ to obtain a CHCl_3_ solution. Unlike the workup in the synthesis of **15** from **14** in Scheme 2, the CHCl_3_ solution was directly evaporated under reduced pressure without washing and drying, at lower temperature at first to remove CHCl_3_ and further at 90 °C to remove remaining H_2_O/DMF, to obtain a CHCl_3_ extract. This extract was separated by column chromatography to give methyl 2-*O*-benzyl-*α*-d-glucopyranoside (**26**) in 46.3% yield. In contrast to the synthesis of **15** from **14** in Scheme 2, the present post-processing in the presence of remaining NaOH in H_2_O/DMF at 90 °C resulted in the hydrolysis of 3-*O*-benzyl and 4,6-*O*-benzylidene groups to produce **26**. Then, reaction of **26** with BnG-Cl (**13**, in Scheme 1) in a 1:2.4 molar ratio in anhydrous pyridine at 60 °C for 48 h produced two di-*O*-esterified products, 4,6-di-*O*-esterified **27** (31.0% yield) and 3,6-di-*O*-esterified **28** (56.3% yield) obtained by chromatographic separation, while reaction of **26** with BnG-Cl in a 1:5.6 molar ratio at the same conditions gave a 3,4,6-tri-*O*-esterified product **29** in 76.2% yield (Scheme 7). Hydrogenation of **27**, **28** and **29** over 10% Pd-C in THF containing suitable amount of aqueous 95% EtOH under 10 atmospheric pressure at 40 °C for 12 h afforded **8**, **9** and **10** in 37.0%, 20.4% and 80.0% yields, respectively.

*Reagents and conditions*: (a) PhCH_2_Br, NaH, DMF, rt, 6 h; (b) BnG-Cl, in anhydrous pyridine, 60 °C, 48 h; (c) H_2_, 10% Pa-C, in THF containing approximately 16% of aqueous 95% EtOH for **27**/**28** and 9% of aqueous 95% EtOH for **29**, 40 °C, 10 atm, 12 h.

#### 2.1.7. Synthesis of **11** and **12** from the Intermediate **17**

**Scheme 8:**
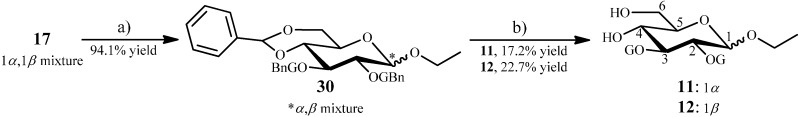
Synthesis of ethyl 2,3-di-*O*-galloyl-*α*-d-glucopyranoside (**11**) and ethyl 2,3-di-*O*-galloyl-*β*-d-glucopyranoside (**12**) from **17**.

Ethyl 2,3-di-*O*-galloyl-*α*-d-glucopyranoside (**11**) and ethyl 2,3-di-*O*-galloyl-*β*-d-glucopyranoside (**12**), were synthesized using **17** (in Scheme 2) as starting materials, as shown in Scheme 8.

*Reagents and conditions*: (a) BnG-Cl, in anhydrous pyridine, 60 °C, 48 h; (b) H_2_, 10% Pa-C, in THFcontaining 6.7% of aqueous 95% EtOH, 40 °C, 10 atm, 12 h.

As shown in Scheme 8, reaction of **17** (1*α*,1*β* mixture, in Scheme 2) with BnG-Cl (**13**, in Scheme 1) in 1:2.3 molar ratio in anhydrous pyridine at 60 °C for 48 h gave 2,3-di-*O*-esterified product **30** (1*α*,1*β* mixture) in 94.1% yield, and hydrogenation of **30** over 10% Pd-C in THF containing 6.7% of aqueous 95% EtOH under 10 atmospheric pressure at 40 °C for 12 h, followed by chromatographic and HPLC separation, afforded **11** and **12** in 17.2% and 22.7% yields, respectively.

### 2.2. Antitumor Activity Evaluation

#### 2.2.1. The *in Vitro* Antitumor Activity of **1–12**

The *in vitro* antitumor activity of **1–12** was assayed by the MTT method on human cancer K562, HL-60 and HeLa cell lines, using 5-flurouracil (5-FU) and docetaxol (DOC) as positive controls at first. In the MTT assay, **1–12**, 5-FU and DOC inhibited the tested three human cancer cell lines by the inhibition rates (IR%) at 100 μg/mL shown in Table 1, and the half inhibitory concentration (IC_50_) of **1–12** was determined as given in Table 2. We then further tested the inhibitory effect of **1–12** on the murine sarcoma S180 cell line by the same MTT assay using taxol as positive control. In the test, **1–12** and taxol inhibited the S180 cells with following IR% values at 100 μg/mL: **1**, 40.7%; **2**, 52.8%; **3**, 39.2%; **4**, 48.7%; **5**, 38.4%; **6**, 47.7%; **7**, 35.7%; **8**, 46.6%; **9**, 40.1%; **10**, 52.4%; **11**, 38.7%; **12**, 47.3%; taxol, 59.1%.

**Table 1:** Inhibition rate (IR%) of **1–12** on human cancer K562, HL-60 and HeLa cells at 100 μg/mL. ^a^

Cells	IR% at 100 μg/mL													
	1	2	3	4	5	6	7	8	9	10	11	12	5-FU	DOC
K562	78.5	70.5	77.0	72.9	70.9	71.5	81.6	65.5	88.1	73.3	74.9	64.2	74.1	71.8
HeLa	86.6	83.7	77.7	85.3	75.5	84.7	88.8	88.8	76.9	78.5	85.6	77.7	90.6	82.8
HL-60	89.4	92.1	92.9	91.8	88.0	89.0	89.0	88.9	89.3	90.6	92.0	88.5	86.3	57.1

Notes: ^a^ The cells were treated with the test samples at the 100 μg/mL for 48 h, and then the inhibition rate (IR%) was determined by the MTT method. 5-FU: 5-flurouracil, DOC: docetaxol.

**Table 2:** Half inhibitory concentration (IC_50_) of **1–12** on human cancer K562, HL-60 and HeLa cells. ^a^

Cells	IC_50_ (μM)											
	1	2	3	4	5	6	7	8	9	10	11	12
K562	77.9	68.2	71.1	115.3	70.3	85.8	77.5	124.7	72.1	49.8	91.0	109.4
HeLa	61.8	44.0	93.8	63.0	52.2	45.3	71.3	66.9	77.3	49.4	68.4	81.3
HL-60	36.2	18.7	35.3	30.8	19.0	19.3	30.9	30.5	39.0	17.2	32.6	32.4

Note: ^a^ The IC_50_ values in this table were determined by the MTT method after treatment of the cells with the test compounds at different concentrations for 48 h.

#### 2.2.2. The *in Vivo* Antitumor Activity of **1** and **2** in Mice

The *in vivo* antitumor activity of **1** and **2** was tested on the murine sarcoma S180 tumor-bearing Kunming mice, and taxol was used as positive control. Compounds **1–2** at 15 and 30 mg/kg dosages and taxol at 20 mg/kg dosage were administered by intravenous injection via the mouse tail vein every day for six continuous days for **1–2** and every other day for six days for taxol, respectively.

On the third day of the last administration, the mice were sacrificed, and the body and tumor weights were weighed. Then, the inhibition rate (IR%) of the test and taxol groups on the tumor growth was calculated using mean tumor-weight (TW) values by the following formula:

IR% = TW_model group_ − TW_sample group_/TW_model group_ × 100%

In the present test, both **1** and **2** at the 30 mg/kg dosage could significantly inhibit the tumor growth, although their inhibitory effect was a little weaker than the effect of the positive control taxol (Table 3), while **1** and **2** at 15 mg/kg dosage did not show significant inhibitory effects on the tumor growth as shown in Table 3. Moreover, in contrast to the significantly reduced body weight of the mice in taxol group, **1** and **2** both at 15 and 30 mg/kg dosages did not significantly affect the body weight of tested mice (Table 3).

**Table 3:** Inhibitory effect of **1** and **2** on the S180 tumor growth in mice (mean ± S.D., *n* = 8 or 11).

Group	Dose (mg/kg)	Body Weight (g)	Tumor Weight (g)	Inhibition Rate (%)
Model group	—	29.54 ± 3.61	2.45 ± 0.79	—
Taxol	20	22.28 ± 2.02 *	1.49 ± 0.46 *	39.2
**1**	15	28.06 ± 3.09	2.10 ± 0.46	14.3
	30	26.90 ± 2.95	1.79 ± 0.45 **	26.9
**2**	15	28.63 ± 3.90	2.41 ± 0.72	1.6
	30	27.22 ± 3.41	1.87 ± 0.41 **	23.7

Notes: * *p* < 0.01, ** *p* < 0.05, compared with model group; *n* = 8 for taxol group and *n* = 11 for other groups.

### 2.3. Discussion

We have presented the synthesis of twelve galloyl glucosides with two (**1**, **3**, **4**, **7–9**, **11** and **12**) and three (**2**, **5**, **6** and **10**) galloyl groups showing diverse substitution patterns, including six with ester glycoside bonds (compounds **1–6**) and other six with general glycoside bonds (compounds **7–12**) covering both *β* (**1–6**) and *α* (**7–12**) configurations. Generally, *α*-anomers of galloyl glucosides are rare in Nature, and because of their lack of availability, studies of *α*-anomers have been very rare and largely lagged compared to the *β*-anomers. Even so, in recently developed studies on *α*-PGG, made possible by chemical synthesis, have shown its unique biological properties [17,21]. In view of this development, we synthesized both *α*,*β*-anomers in the present study. Among the twelve synthesized galloyl glucosides, **9**, **11** and **12** are new compounds, and **1–4**, **6** and **10** were synthesized for the first time in the present study, although some galloyl glucosides, including **1–2** [44], **3** [45], **4** [46], **5** [47], **6** [48] and the C-1 epimer of **9** (methyl 3,6-di-*O*-galloyl-*β*-d-glucopyranoside [49]), had been isolated from plants [44,45,46,47,48,49] and some of the galloyl glucosides, including **5** [28], **7** [26] and **8** [5], had also been synthesized [5,25,26,27,28,29]. In [4] an isolated galloyl glucoside was recorded as methyl 3,6-di-*O*-galloyl-*α*-d-glucopyranoside (**9**) in the Abstract and Section 2.1, but the structure of the compound given in the text, coupled with the cited literature [49] for identification without data, indicated that the name of this compound in the literature [4] was a miswriting of the correct name methyl 3,6-di-*O*-galloyl-*β*-d-glucopyranoside [49], and no more reports so far have mentioned **9**. In addition, **8** has also once been reported to be synthesized [5], but no any supporting data were provided, and the physicochemical and spectroscopic data of **8** were recorded for the first time in the present paper.

It is noteworthy that in the 4,6-*O*-benzylidende ring forming reaction we used triethyl orthoformate for the first time for the *p*TSA-catalyzed reaction of d-glucose and benzaldehyde, as shown in Scheme 2, to obtain the 4,6-*O*-benzylidende protected ethyl glycoside **17** by a one-pot reaction. Under the acidic reaction conditions, the triethyl orthoformate underwent hydrolysis to produce EtOH, which in turn further reacted with the reactive 1-OH in glucose, eventually affording **17**. In contrast, the 1-OH free, 4,6-*O*-protected product **16** could be obtained by the same *p*TSA-catalyzed reaction of the d-glucose and benzaldehyde without triethyl orthoformate (Scheme 2). Generally, triethyl orthoformate was used for the purpose of dehydration in relevant reactions by its hydrolysis to produce ethyl formate or formic acid and EtOH. Triethyl orthoformate has also been used in the *p*TSA-catalyzed reaction of a *β*-d-glucoside and benzaldehyde for protecting the 4,6-OH groups in glucose and the target 4,6-*O*-benzylidende-protected product with an unchanged *β*-d-glycoside bond was obtained in [50]. Similarly, the *p*TSA-catalyzed reaction of methyl *α*-d-glucoside and benzaldehyde in the presence of triethyl orthoformate in the present study exclusively produced the unchanged *α*-glycoside bond 4,6-*O*-benzylidende protected product **14** (Scheme 2), indicating the stability of the methyl *α*-glycoside bond under the acidic reaction conditions. Under the acidic reaction conditions, triethyl orthoformate should undergo hydrolysis (by H_2_O from the reaction of methyl *α*-d-glucoside and benzaldehyde) to produce formic acid and EtOH. If the methyl *α*-d-glucoside were also hydrolyzed, the reactive 1-OH in the d-glucose produced should further react with EtOH to also produce an ethyl 4,6-*O*-benzylidene-d-glucoside as byproduct, as seen in the synthesis of **17**.

The hydrolysis-resistant nature of the methyl *α*-d-glycoside bond was also indicated by the acid-catalyzed hydrolysis of **15**. In the hydrolysis of **15** by 2 N HCl at 80 °C for 16 h, shown in Scheme 3, in addition to the hydrolyzed product **18**, quite a large amount of the 1-OCH_3_ unhydrolyzed material methyl 2,3-di-*O*-benzyl-*α*-d-glucopyranoside was produced in every round of hydrolysis, and the 41.2% total yield of **18** was obtained by repeated hydrolyses of the 1-OCH_3_ unhydrolyzed materials. Incidentally, later by an additional hydrolysis of **15** using 30% trifluoroacetic acid (TFA) as catalyst in CH_3_CN-TFA-H_2_O (3:3:4) at 98 °C for 48 h according to the method in the literature [51], we could obtain **18** in quite a high 40% yield through a one-step hydrolysis.

In the *in vitro* MTT assay, compounds **1–12** inhibited the human cancer K562, HL-60 and HeLa cells with inhibition rates ranging from 64.2% to 92.9% at 100 μg/mL (Table 1), and their IC_50_ values were determined to vary from 17.2–124.7 μM on the tested cancer cell lines (Table 2). In the *in vitro* MTT assay, **1–12** also inhibited the murine sarcoma S180 cells with inhibition rates ranging from 38.7% to 52.8% at the 100 μg/mL (see Section 2.2.1). The MTT assay for the synthesized **1** and **2** on K562 cells (IC_50_: 77.9 μM for **1** and 68.2 μM for **2**) reconfirmed our assay previously reported result for the natural products (IC_50_: 80.8 μM for natural **1** and 64.8 μM for natural **2**) [44]. In addition, it seems from the IC_50_ values in Table 2 that the galloyl glucosides with three galloyl groups (**2**, **5**, **6** and **10**) possess stronger inhibitory activity than those with two galloyl groups (**1**, **3**, **4**, **7–9**, **11** and **12**) on the tested three human cancer cell lines, except for **6** on the K562 cell line, and further on the whole, **1–12** all showed stronger activity on the HL-60 cells than on the K562 and HeLa cells (Table 2). Moreover, two pairs of the *α*,*β*-anomers, **5**/**6** and **11**/**12**, showed no difference in their inhibition of tested human cancer cell lines between the corresponding *α*,*β*-anomers (Table 2), and no relationship between inhibitory activity and substitution patterns in **1–12** could be found from the IC_50_ values given in Table 2. We then carried out antitumor tests in mice for **1** and **2**, which were obtained in a larger amount for the test, *in vivo* antitumor activity of **1** and **2** was detected on the murine sarcoma S180 tumor-bearing Kunming mice using taxol as positive control as shown in Table 3. The same S180 tumor-bearing mice model were also used in [52,53] for an *in vivo* antitumor test by a similar procedure [52,53], including the use of taxol as positive control [52]. Although the *in vivo* antitumor activity of **1** and **2** in the present test was certainly weaker than that of taxol, it is noteworthy that **1** and **2** showed also very weak toxicity to the host, as indicated by body weight changes of the tested mice (Table 3), as documented for PGG in [18,20]. In view of the present results, it seems worthy to further evaluate the *in vivo* antitumor activity of these galloly glucosides in detail, including whether they would exert preventive effect on the tumor development in mice by pre-administration, and so on.

## 3. Experimental Section

### 3.1. General

Melting points were measured on a Beijing Tiandiyu X-4 exact micro melting point apparatus (Tiandiyu Science and Technology Co., Ltd., Beijing, China) and the temperature was not corrected. Optical rotations were measured on an Optical Activity Limited polAAr 3005 spectropolarimeter (Optical Activity Limited, Ramsey, UK). ESIMS was recorded on an Applied Biosystems API 3000 LC-MS spectrometer (AB SCIEX, Framingham, MA, USA). ^1^H- and ^13^C-NMR spectra were obtained on a JEOL JNM-GX 400 (400 MHz ^1^H- and 100 MHz ^13^C-NMR) NMR spectrometer (JEOL Ltd., Tokyo, Japan). The chemical shifts of ^1^H- and ^13^C-NMR signals were recorded in δ values using the solvent signals (CDCl_3_: δ_H_ 7.26/δ_C_ 77.1; CD_3_OD: δ_H_ 3.31/δ_C_ 49.0) as references, respectively. A VERSAmax-BN03152 micro plate reader (Molecular Devices, Silicon Valley, CA, USA) was used to read the optical density (OD) and an AE31 EF-INV inverted microscope (Motic China Group Co., Ltd., Xiamen, Fujian, China) was used for examination of tumor cell morphology.

Precoated silica gel GF_254_ plates (10 cm × 20 cm, 0.25 mm thickness, Yantai Chemical Industrial Institute, Yantai, China) and polyamide thin layers (10 cm × 20 cm, Taizhou Luqiao Sijia Biochemical Plastic Factory, Taizhou, China) were used in TLC, and spots were detected under UV light (254 and 365 nm) or by using 10% sulfuric acid reagent, Vaughan’s reagent [24 g of ammonium molybdate tetrahydrate (NH_4_)_6_Mo_7_O_24_·4H_2_O and 1 g of ceric sulfate Ce(SO_4_)_2_ dissolved in 500 mL of 10% H_2_SO_4_], or 5% FeCl_3_ reagent (5 g of FeCl_3_ dissolved in 100 mL of 95% aqueous EtOH). Polyamide (100–200 mesh, Taizhou Luqiao Sijia Biochemical Plastic Factory) and Sephadex™ LH-20 (GE Healthcare, Uppsala, Sweden) were used for column chromatography. HPLC was carried out on a Waters HPLC system (Waters, Milford, MA, USA) equipped with Waters 600 controller, Waters 600 pump, Waters 2996 (for analytical) or 2998 (for preparative) photodiode array detector, and Waters Empower™ software. The Capcell Pak C18 columns (MG II S5, 4.6 × 250 mm and 20 × 250 mm; Shiseido Co., Ltd., Tokyo, Japan) were used in analytical and preparative HPLC, respectively.

The human chronic myelogenous leukemia K562 cell line was provided by Prof. Dr. Lili Wang (Beijing Institute of Pharmacology and Toxicology, Beijing, China). Human acute promyelocytic leukemia HL-60 and human cervical cancer HeLa cell lines were provided by Prof. Dr. Wenxia Zhou (Beijing Institute of Pharmacology and Toxicology). Fetal bovine serum was purchased from Tianjin Hao Yang Biological Manufacture Co., Ltd. (Tianjin, China). The RPMI-1640 medium (lot No. 1403238) was purchased from Gibco (Grand Island, NY, USA) and MTT (lot No. 0793) from Amresco (Solon, OH, USA). Streptomycin (lot No. 121104) and penicillin (lot No. X1303302) were purchased from North China Pharmaceutical Group Corporation (Beijing, China). The 5-fluorouracil (5-FU, lot No.5402), docetaxol (DOC, lot No.20110326) and taxol (lot No.20110427) were purchased from Aladdin Chemistry Co. Ltd. (Shanghai, China). Gallic acid (lot No. F20070926) and d-glucose (lot No. 20110514) were purchased form Sinopharm Chemical Reagent Co., Ltd. (Beijing, China). All other chemicals were purchased as the reagent grade and used without purification unless otherwise noted. Benzaldehyde was distilled and pyridine was dried by KOH for 24 h, before use in reactions. The petroleum ether with boiling point 60–90 °C was used throughout the experiments and was abbreviated as Pet.Et_2_O in the following descriptions.

### 3.2. Chemical Synthesis

#### *1,6-Di-O-galloyl-β-d-glucopyranose* (**1**)

A solution of **15** (17.4 g, 0.0376 mol) in aqueous 2 N HCl (640 mL) was refluxed at 80 °C for 16 h to hydrolyze **15**. The reaction mixture was then extracted with EtOAc (300 mL × 4) to give an EtOAc extract. The EtOAc extract was separated by column chromatography on silica gel (bed, 5.0 × 30.0 cm; eluted by CH_2_Cl_2_-MeOH 30:1) to obtain hydrolyzed product **18** and quite a larger amount of the 1-OCH_3_ unhydrolyzed materials, methyl 2,3-di-*O*-benzyl-*α*-d-glucopyranoside. The latter was further subjected, without weighing, to the same hydrolysis and separation steps to obtain additional amounts of **18**, and the recovered raw materials in this round of experiments were also hydrolyzed and then separated once again to obtain further additional amount of **18**. Eventually, **18** (5.6 g, 0.0155 mol, in 41.2% yield) was obtained from 17.4 g (0.0376 mol) of **15** as an amorphous powder in CH_2_Cl_2_-MeOH. Totally over 10 g of **18** were prepared from **15** by additionally repeated hydrolyses. 2,3-Di*-O-*benzyl-d-glucopyranose (**18**): An amorphous powder (CH_2_Cl_2_-MeOH), positive ion ESI-MS *m/z*: 361 [M+H]^+^, 383 [M+Na]^+^; negative ion ESI-MS *m/z*: 405 [M+HCO_2_]^−^. ^1^H-NMR (400 MHz, CDCl_3_) *δ* for *α*-isomer: 7.39–7.21 (10H, m, Ph-H_5_ in PhCH_2_ × 2), 5.21 (1H, d, *J* = 3.6 Hz, H-1), 4.85 and 4.84 (each 1H, d, *J* = 11.1 Hz, PhCH_2_), 4.69 and 4.65 (each 1H, d, *J* = 11.9 Hz, PhCH_2_), 3.81–3.75 (1H, m, H-5), 3.79 (1H, t, *J* = 9.5 Hz, H-3), 3.77 (1H, dd, *J* = 12.2, 2.2 Hz, H_a_-6), 3.68 (1H, dd, *J* = 12.2, 5.3 Hz, H_b_-6), 3.45 (1H, t, *J* = 9.5 Hz, H-4), 3.42 (1H, dd, *J* = 9.5, 3.6 Hz, H-2); *δ* for *β*-isomer: 7.39–7.21 (10H, m, Ph-H_5_ in PhCH_2_ × 2), 4.94 and 4.80 (each 1H, d, *J* = 11.2 Hz, PhCH_2_), 4.86 and 4.68 (each 1H, d, *J* = 11.2 Hz, PhCH_2_), 4.64 (1H, d, *J* = 8.0 Hz, H-1), 3.86 (1H, dd, *J* = 11.9, 2.2 Hz, H_a_-6), 3.65 (1H, dd, *J* = 11.9, 5.3 Hz, H_b_-6), 3.46 (1H, t, *J* = 9.5 Hz, H-4), 3.43 (1H, t, *J* = 9.5 Hz, H-3), 3.34–3.29 (1H, m, H-5), 3.23 (1H, dd, *J* = 9.5, 8.0 Hz, H-2). ^13^C-NMR (100 MHz, CDCl_3_) *δ* for *α*-isomer: 140.7, 140.0, 129.5–128.7 (10C), all Ph-C_6_ in PhCH_2_ × 2; 92.1 (C-1), 83.0 (C-3), 81.5 (C-2), 76.7 (PhCH_2_), 73.9 (PhCH_2_), 73.2 (C-5), 71.9 (C-4), 62.7 (C-6); *δ* for *β*-isomer: 140.5, 140.3 129.5–128.7 (10C), all Ph-C_6_ in PhCH_2_ × 2; 98.8 (C-1), 86.2 (C-3), 84.6 (C-2), 78.1 (C-5), 76.1 (PhCH_2_), 75.9 (PhCH_2_), 72.0 (C-4), 63.0 (C-6).

A mixed solution of **18** (3.0 g, 0.0083 mol) and **13** (10.3 g, 0.0225 mol) in 30 mL of anhydrous pyridine was stirred at 60 °C for 48 h and then evaporated under reduced pressure to obtain a reaction mixture. The reaction mixture was separated by silica gel column chromatography (bed, 3.5 × 15.0 cm; eluted by Pet.Et_2_O-acetone 20:1) to afford crude **19** (8.0 g, 0.0066 mol, 79.5% yield), which did not give *pseudo*-molecular ion peaks in both positive and negative ESI-MS measurements. Crude **19** (8.0 g, 0.0066 mol) was dissolved in 200 mL of THF-95% EtOH (15:5) solution, transferred into a 0.5 L high-pressure reactor, and then reduced by H2 under 10 atmospheric pressure at 40 °C for 12 h using 10% Pd-C (0.8 g) as catalyst. The reaction mixture was filtered to filter out insoluble materials and evaporated under reduced pressure to give a reaction product. This product was separated by repeated Sephadex LH-20 column chromatography eluted with H_2_O-MeOH (75:25→25:75) to afford crude **1**. The crude **1** was recrystallized in 30% MeOH to obtain pure **1** (1.2 g, 0.0025 mol, yield 37.9%). Needles (MeOH-H_2_O), m.p. 180–182 °C; ${\left[\alpha \right]}_{\text{D}}^{25}$ −19.4 (*c* 0.52, MeOH); positive ion ESI-MS *m/z*: 507 [M+Na]^+^; negative ion ESI-MS *m/z*: 483 [M−H]^−^. ^1^H- and ^13^C-NMR: Table 4 and Table 5.

**Table 4:** 400 MHz ^1^H-NMR data of **1–12** in CD_3_OD ^a^.

**Position**	***δ*_H_**					
	**1**	**2**	**3**	**4**	**5**	**6**
glucose						
1	5.69 (d, *J* = 6.3 Hz)	5.79 (d, *J* = 8.3 Hz)	5.90 (d, *J* = 8.6 Hz)	5.79 (d, *J* = 8.2 Hz)	6.06 (d, *J* = 8.3 Hz)	6.60 (d, *J* = 3.5 Hz)
2	3.56–3.49 (m)	3.66 (dd, *J* = 9.5, 8.3 Hz)	5.19 (t, *J* = 8.6 Hz)	3.79–3.68 (m)	5.42 (dd, *J* = 9.7, 8.3 Hz)	5.29 (dd, *J* = 10.1, 3.5 Hz)
3	3.56–3.49 (m)	3.85 (t, *J* = 9.5 Hz)	3.82–3.70 (m)	5.25 (t, *J* = 9.4 Hz)	5.54 (t, *J* = 9.7 Hz)	5.86 (t, *J* = 10.1 Hz)
4	3.56–3.49 (m)	5.23 (t, *J* = 9.5 Hz)	3.58–3.52 (m)	3.79–3.68 (m)	3.88 (t, *J* = 9.7 Hz)	4.01–3.91 (m)
5	3.76–3.67 (m, 1H)	4.07 (ddd, *J* = 9.5, 4.6, 2.0 Hz)	3.58–3.52 (m)	3.61–3.53 (m)	3.70 (ddd, *J* = 9.7, 4.6, 2.2 Hz)	4.01–3.91 (m)
6	4.55 (br d, *J* = 12.0 Hz)	4.45 (dd, *J* = 12.4, 2.0 Hz)	3.90 (br d, *J* = 11.9 Hz)	3.88 (br d, *J* = 12.1 Hz)	3.93 (dd, *J* = 12.3, 2.2 Hz)	3.90–3.78 (2H, m)
	4.40 (dd, *J* = 12.0, 4.5 Hz)	4.22 (dd, *J* = 12.4, 4.7 Hz)	3.82–3.70 (m)	3.79–3.68 (m)	3.81 (dd, *J* = 12.3, 4.6 Hz)	
galloyl						
2,6	7.13 (2H, s)	7.15 (2H, s)	7.04 (2H, s)	7.15 (2H, s)	7.03 (2H, s)	7.17 (2H, s)
	7.08 (2H, s)	7.11 (2H, s)	7.01 (2H, s)	7.12 (2H, s)	7.02 (2H, s	7.06 (2H, s)
		7.07 (2H, s)			6.92 (2H, s)	6.91 (2H, s)
**Position**	***δ*_H_**					
	**7**	**8**	**9**	**10**	**11**	**12**
glucose						
1	5.05–4.98 (m)	4.77 (d, *J* = 3.6 Hz)	4.77 (d, *J* = 3.7 Hz)	4.86 (d, *J* = 3.7 Hz)	5.15 (d, *J* = 3.6 Hz)	4.73 (d, *J* = 8.0 Hz)
2	5.05–4.98 (m)	3.58 (dd, *J* = 9.5, 3.6 Hz)	3.71 (dd, *J* = 9.9, 3.7 Hz)	3.88 (dd, *J* = 9.9, 3.7 Hz)	4.99 (dd, *J* = 10.2, 3.6 Hz)	5.08 (dd, *J* = 9.8, 8.0 Hz)
3	5.66 (t, *J* = 8.3 Hz)	3.93 (t, *J* = 9.5 Hz)	5.38 (t, *J* = 9.9 Hz)	5.64 (t, *J* = 9.7 Hz)	5.68 (dd, *J* = 10.2, 8.6 Hz)	5.39 (dd, *J* = 9.8, 9.6 Hz)
4	3.82–3.70 (m)	5.10 (t, *J* = 9.5 Hz)	3.66 (t, *J* = 9.9 Hz)	5.35 (t, *J* = 9.7 Hz)	3.91–3.70 (m)	3.74 (t, *J* = 9.6 Hz)
5	3.82–3.70 (m)	4.11–4.05 (m)	3.95 (ddd, *J* = 9.9, 5.8, 2.0 Hz)	4.27–4.20 (m)	3.91–3.70 (m)	3.51 (ddd, *J* = 9.6, 5.3, 2.2 Hz)
6	3.89 (br d, *J* = 11.6 Hz)	4.38 (br d, *J* = 11.8 Hz)	4.55 (dd, *J* = 11.9, 2.0 Hz)	4.43 (br d, *J* = 10.3 Hz)	3.91–3.70 (2H, m)	3.77 (dd, *J* = 12.1, 5.3 Hz)
	3.82–3.70 (m)	4.19 (dd, *J* = 11.8, 5.8 Hz)	4.41 (dd, *J* = 11.9, 5.8 Hz)	4.30–4.24 (m)		3.98–3.89 (m)
galloyl						
2,6	7.03 (2H, s)	7.09 (2H, s)	7.13 (2H, s)	7.08 (2H, s)	7.03 (2H, s)	7.00 (2H, s)
	6.98 (2H, s)	7.07 (2H, s)	7.09 (2H, s)	6.99 (2H, s)	6.98 (2H, s)	6.96 (2H, s)
			6.95 (2H, s)		
OCH_3_	3.44 (3H, s)	3.46 (3H, s)	3.48 (3H, s)	3.52 (3H, s)	—	—
OCH_2_CH_3_	—	—	—	—	3.91–3.70 (1H, m)	3.98–3.89 (1H, m)
					3.52 (1H, dq, *J* = 10.3, 7.1 Hz)	3.61 (1H, dq, *J* = 9.7, 7.1 Hz)
OCH_2_CH_3_	—	—	—	—	1.23 (3H, t, *J* = 7.1 Hz)	1.12 (3H, t, *J* = 7.1 Hz)

Notes: ^a^ Chemical shifts were recorded in *δ*_C_ values using the solvent signal (CD_3_OD: *δ*_H_ 3.31) as reference. Signal assignments were based on the results of ^1^H-^1^H COSY and HMQC experiments.

**Table 5:** 100 MHz ^13^C-NMR data of **1–12** in CD_3_OD ^a^.

Position	*δ*_C_											
	1	2	3	4	5	6	7	8	9	10	11	12
glucose												
1	95.9	95.8	94.1	95.8	93.8	91.2	98.5	101.1	101.2	101.3	97.2	102.0
2	74.0	74.2	74.3	72.6	72.3	71.9	73.1	73.5	71.9	71.7	73.1	73.5
3	78.0	75.9	76.1	79.1	76.7	74.0	74.2	73.0	76.9	74.4	74.3	77.0
4	71.1	71.7	71.2	69.3	69.2	69.0	69.8	72.6	70.2	70.6	69.9	69.8
5	76.4	74.3	79.0	78.7	78.9	76.5	73.6	69.2	71.2	69.2	73.6	78.0
6	64.4	63.5	62.2	61.9	61.8	61.8	62.2	64.1	64.7	63.8	62.2	62.3
galloyl												
CO	168.3	168.0	167.6	168.1	167.7	168.1	168.2	168.1	168.35	168.0	168.2	167.8
	167.0	167.4	166.5	168.1	167.1	167.3	167.6	167.5	168.25	167.9	167.7	167.2
		166.8			166.3	166.3				167.2		
1	121.2	121.1	121.1	121.6	121.0	121.1	121.3	121.2	121.7	121.1 (2C)	121.3	121.1
	120.5	120.9	120.1	120.5	120.4	120.4	120.5	121.0	121.3	120.4	120.6	121.0
		120.4			119.9	120.2						
2,6	110.5	110.5	110.4	110.5	110.5	110.42	110.34	110.3	110.3	110.34	110.32	110.3
	110.1	110.3	110.3	110.3	110.35	110.35	110.28	110.1	110.0	110.31	110.27	110.2
		110.2			110.28	110.33				110.17		
3,5	146.48	146.49	146.46	146.5	146.5	146.7	146.37	146.5 (4C)	146.5	146.5	146.4	146.33
	146.44	146.44	146.37	146.4	146.31	146.4	146.34		146.3	146.4	146.3	146.28
		146.37			146.29	146.3				146.3		
4	140.4	140.4	140.5	140.4	140.6	140.6	140.1	140.0	139.9	140.2	140.0	139.9
	139.9	140.0	140.0	139.7	140.1	140.2	139.8	139.8	139.7	139.9	139.8	139.8
		139.8			139.9	140.0				139.8		
OCH_3_	—	—	—	—	—	—	55.7	55.8	55.7	56.0	—	—
OCH_2_CH_3_	—	—	—	—	—	—	—	—	—	—	64.7	66.4
OCH_2_CH_3_	—	—	—	—	—	—	—	—	—	—	15.4	15.5

Notes: ^a^ Chemical shifts were recorded in *δ*_C_ values using the solvent signal (CD_3_OD: *δ*_C_ 49.00) as reference. Signal assignments were based on the results of ^1^H-^1^H COSY and HMQC experiments.

#### *1,4,6-Tri-O-galloyl-β-d-glucopyranose* (**2**)

A mixed solution of **18** (4.5 g, 0.0125 mol) and **13** (42.0 g, 0.0917 mol) in anhydrous pyridine (170 mL) was stirred at 60 °C for 48 h and then evaporated under reduced pressure to obtain a reaction mixture. The mixture was subjected to silica gel column chromatography eluted with CH_2_Cl_2_-EtOAc (100:1) to afford crude **20** (15.5 g, 0.0095 mol, 76.0% yield), which did not give the corresponding *pseudo*-molecular ion peaks in positive and negative ESI-MS measurements. The crude **20** (15.5 g, 0.0095 mol) was dissolved in THF-95% EtOH (17:6, 230 mL), transferred into a 1 L high-pressure reactor, and reduced by H_2_ under 10 atmospheric pressure at 40 °C for 12 h using 10% Pd-C (1.0 g) as catalyst. The reaction mixture was filtered to filter out insoluble materials and evaporated to give a reaction product. This product was separated by repeated Sephadex LH-20 column chromatography eluted with H_2_O-EtOH (45:55) to afford crude **2**, which was recrystallized in 40% MeOH to give pure **2** (2.3 g, 0.0036 mol, yield 37.9%). Needles (MeOH-H_2_O), m.p. 173–175 °C; ${\left[\alpha \right]}_{\text{D}}^{25}$ +50.2 (*c* 0.51, MeOH); positive ion ESI-MS *m/z*: 656 [M+Na]^+^; negative ion ESI-MS *m/z*: 635 [M−H]^−^, 671 [M+Cl]^−^. ^1^H- and ^13^C-NMR: Table 4 and Table 5.

#### *1,2-Di-O-galloyl-β-d-glucopyranose* (**3**), *1,3-di-O-galloyl-β-d-glucopyranose* (**4**), *1,2,3-tri-O-Galloyl-β-d-glucopyranose* (**5**) *and 1,2,3-tri-O-galloyl-α-d-glucopyranose* (**6**)

A mixed solution of **16** (2.0 g, 0.0075 mol) and **13** (8.5 g, 0.0186 mol) in anhydrous pyridine (40 mL) was stirred at 60 °C for 48 h and then evaporated under reduced pressure to obtain a reaction mixture that was separated by silica gel column chromatography (bed, 3.0 × 13.0 cm) using CH_2_Cl_2_ as eluting solvent to afford a mixture of three esterified products **21** (5.8 g). The mixture was dissolved in THF-95% EtOH (14:1, 150 mL), transferred into a 0.5 L high-pressure reactor, and then reduced by H_2_ under 10 atmospheric pressure at 40 °C for 12 h using 10% Pd–C (0.7 g) as catalyst. The reaction mixture was filtered to filter out the undissolved materials and evaporated under reduced pressure to obtain a reaction product. This reaction product was separated by Sephadex LH-20 column (bed, 1.8 × 60.0 cm; eluted by H2O-EtOH 60:40) to afford **4** (150 mg, 0.31 mmol, 4.1% yield from **16**) and fractions **Fr-2** and **Fr-3**. Further separation of **Fr-2** by preparative HPLC (Capcell Pak C18 column, MGII S5, 20 × 250 mm; 20% methanol, 10 mL/min) gave **5** (200 mg, 0.31 mmol, *t*_R_ = 37.0 min, 4.1% yield from **16**) and **6** (100 mg, 0.16 mmol, *t*_R_ = 50.0 min, 2.1% yield from **16**), while HPLC separation of **Fr-3** at the same conditions, except for the use of 10% methanol as mobile phase, afford **3** (50 mg, 0.11 mmol, *t*_R_ = 60.0 min, 1.5% yield from **16**). *1,2-Di-O-galloyl-β-d-glucopyranose* (**3**): A crystalline powder (MeOH), m.p. 150–153 °C; ${\left[\alpha \right]}_{\text{D}}^{25}$ −64.5 (*c* 0.44, MeOH); positive ion ESI-MS *m/z*: 502 [M+NH_4_]^+^, 507 [M+Na]^+^; negative ion ESI-MS *m/z*: 483 [M−H]^−^, 519 [M+Cl]^−^. *1,3-Di-O-galloyl-β-d-glucopyranose* (**4**): A crystalline powder (MeOH), m.p. 161–163 °C; ${\left[\alpha \right]}_{\text{D}}^{25}$ +20.4 (*c* 0.44, MeOH); positive ion ESI-MS *m/z*: 507 [M+Na]^+^; negative ion ESI-MS *m/z*: 483 [M−H]^−^, 519 [M+Cl]^−^, 529 [M+HCO_2_]^−^. *1,2,3-Tri-O-galloyl-β-d-glucopyranose* (**5**): A crystalline powder (MeOH), m.p. 176–178 °C; ${\left[\alpha \right]}_{\text{D}}^{25}$ +46.7 (*c* 0.48, MeOH); positive ion ESI-MS *m/z*: 654 [M+NH_4_]^+^ 659 [M+Na]^+^; negative ion ESI-MS *m/z*: 635 [M−H]^−^, 671 [M+Cl]^−^, 681 [M+HCO2]^−^. *1,2,3-Tri-O-galloyl-α-d-glucopyranose* (**6**): A crystalline powder (MeOH), m.p. 1165–167 °C; ${\left[\alpha \right]}_{\text{D}}^{25}$ +282.9 (*c* 0.49, MeOH); positive ion ESI-MS *m/z*: 654 [M+NH_4_]^+^, 659 [M+Na]^+^; negative ion ESI-MS *m/z*: 635 [M−H]^−^, 671 [M+Cl]^−^. ^1^H- and ^13^C-NMR data for 3–6: Table 4 and Table 5.

#### *Methyl 2,3-Di-O-galloyl-α-d-glucopyranoside* (**7**)

A mixed solution of **14** (1.5 g, 0.0053 mol) and **13** (10.0 g, 0.0218 mol) in anhydrous pyridine (50 mL) was stirred at 60 °C for 48 h and evaporated under reduced pressure to give a reaction mixture. The reaction mixture was subjected to column chromatography on silica gel (bed: 5.0 × 10.0 cm), eluted by CH_2_Cl_2_, to afford **22** (5.0 g, 0.0044 mol, yield 83.0%). A portion of **22** (3.5 g, 0.0031 mol) was dissolved in 150 mL of THF-95% EtOH (13:2), transferred into a 0.5 L high-pressure reactor, and then reduced by H_2_ under 10 atmospheric pressure at 40 °C for 12 h using 10% Pd-C (1.5 g) as catalyst. The reaction mixture was filtered to filter out insoluble substances and evaporated to give a reaction product containing **23** (positive ion ESI-MS *m/z*: 587 [M+H]^+^, 609 [M+Na]^+^; negative ion ESI-MS *m/z*: 585 [M−H]^−^) as major component, which was further hydrolyzed without purification in 40 mL of aqueous 1 N HCl at 55 °C for 6 h. The hydrolysate was separated by column chromatography on polyamide (bed: 3.0 × 26.0 cm, eluted by 70% EtOH) and then Sephadex LH-20 (bed: 2.8 × 55.0 cm, eluted by 85% MeOH) to afford **7** (1.0 g, 0.0020 mol, 64.5% yield from **22**). A crystalline powder (MeOH), m.p. 160–162 °C; ${\left[\alpha \right]}_{\text{D}}^{25}$ +171.8 (*c* 0.52, MeOH); positive ion ESI-MS *m/z*: 499 [M+H]^+^, 521 [M+Na]^+^; negative ion ESI-MS *m/z*: 497 [M−H]^−^, 533 [M+Cl]^−^. ^1^H- and ^13^C-NMR: Table 4 and Table 5.

#### *Methyl 4,6-Di-O-Galloyl-α-d-glucopyranoside* (**8**) from **15**

A solution of **15** (10 g, 0.0216 mol) in 50% aqueous AcOH (200 mL) was refluxed at 55 °C for 24 h and then evaporated under reduced pressure to give a reaction mixture. The reaction mixture was separated by column chromatography on silica gel (bed: 4.0 × 10.0 cm), eluted by gradient Pet.Et_2_O-CH_2_Cl_2_ (3:1)→CH_2_Cl_2_-MeOH (20:1), to afford methyl *2,3-di-O-benzyl-α-d-glucopyranoside* (**24**), 6.0 g, 0.0160 mol, 74.1% yield from (**15**) as a crystalline powder (CH_2_Cl_2_-MeOH), m.p. 81–83 °C, ${\left[\alpha \right]}_{\text{D}}^{25}$ +19.0 (*c* 1.00, CH_2_Cl_2_); positive ion ESI-MS *m/z*: 397 [M+Na]^+^; negative ion ESI-MS *m/z*: 373 [M−H]^−^, 419 [M+HCO_2_]^−^. ^1^H-NMR (400 MHz, CDCl_3_) *δ*: 7.39–7.28 (10H, m, Ph-H_5_ in PhCH_2_ × 2), 5.02 and 4.71 (each 1H, d, *J* = 11.5 Hz, PhCH_2_), 4.77 and 4.66 (each 1H, d, *J* = 12.1 Hz, PhCH_2_), 4.59 (1H, d, *J* = 3.5 Hz, H-1), 3.83–3.70 (3H, m, H-3 and H_2_-6), 3.61 (1H, dt, *J* = 9.7, 3.9 Hz, H-5), 3.52 (1H, dd, *J* = 9.7, 8.8 Hz, H-4), 3.49 (1H, dd, *J* = 9.6, 3.5 Hz, H-2), 3.38 (3H, s, OCH_3_). ^13^C-NMR (100 MHz, CDCl_3_) *δ*: 138.7, 138.0, 128.8 (2C), 128.6 (2C), 128.3 (2C), 128.2 (2C), 128.1, 128.0, all Ph-C_6_ in PhCH_2_ × 2; 98.3 (C-1), 81.4 (C-3), 79.8 (C-2), 75.5 (PhCH_2_), 73.3 (PhCH_2_), 70.7 (C-5), 70.4 (C-4), 62.5 (C-6), 55.4 (OCH_3_).

A mixed solution of **24** (1.9 g, 0.0051 mol) and **13** (8.0 g, 0.0175 mol) in anhydrous pyridine (30 mL) was stirred at 60 °C for 48 h and then evaporated under reduced pressure to obtain a reaction mixture that was separated by silica gel column chromatography (bed: 3.0 × 15.0 cm, eluted with a gradient of Pet. Et_2_O-CH_2_Cl_2_ 2:1→CH_2_Cl_2_) to afford a crude **25** (5.5 g, 0.0045 mol, 88.2% yield). The crude **25** (5.5 g, 0.0045 mol) was dissolved without further purification in THF-95% EtOH (13:2, 150 mL), transferred into a 0.5 L high-pressure reactor, and then reduced by H_2_ under 10 atmospheric pressure at 40 °C for 12 h using 10% Pd-C (2.0 g) as catalyst. The reaction mixture was filtered to filter out insoluble substances and evaporated under reduced pressure to give a reaction product. This product was separated by Sephadex LH-20 column (bed, 2.8 × 60.0 cm; eluted by 40% EtOH) to give **8** (1.0 g, 0.0020 mol, 44.4% yield). A crystalline powder (MeOH), m.p. 163–165 °C; ${\left[\alpha \right]}_{\text{D}}^{25}$ +113.6 (*c* 0.47, MeOH); positive ion ESI-MS *m/z*: 521 [M+Na]^+^, 537 [M+K]^+^; negative ion ESI-MS *m/z*: 497 [M−H]^−^. ^1^H- and ^13^C-NMR: Table 4 and Table 5.

#### *Methyl 3,6-Di-O-Galloyl-α-d-glucopyranoside* (**9**) and *Methyl 4,6-di-O-Galloyl-α-d-glucopyranoside* (**8**) from **14**

A mixed solution of **14** (85 g, 0.3014 mol) and NaH (25 g) in DMF (1200 mL) was stirred for 40 min, during which time the solution became hot while stirring and was thus cooled down to room temperature. To the mixture under stirring benzyl bromide (200 mL) was added dropwise and reacted at room temperature for 6 h. The reaction mixture solution was poured into distilled water (2000 mL) and extracted with CHCl_3_ (2000 mL × 3) to give a CHCl_3_ solution. Without washing with water and drying by anhydrous MgSO4 to remove remaining NaOH (from NaH) in CHCl_3_, the CHCl_3_ solution was directly evaporated under reduced pressure, at the lower temperature at first to remove CHCl_3_ and further at 90 °C to remove remaining DMF, to obtain a reaction mixture that was separated by silica gel column chromatography (bed, 7.0 × 14.0 cm; eluted by gradient Pet. Et_2_O-EtOAc 10:1→5:1) to afford **26** (40 g, 0.1408 mol, 46.7% yield). *Methyl 2-O-benzyl-α-d-glucopyranoside* (**26**): A crystalline powder (MeOH), m.p. 113–115 °C, ${\left[\alpha \right]}_{\text{D}}^{25}$ +76.5 (*c* 1.00, MeOH); positive ion ESI-MS *m/z*: 302 [M+NH_4_]^+^, 307 [M+Na]^+^, 323 [M+K]^+^; negative ion ESI-MS *m/z*: 283 [M−H]^−^, 319 [M+Cl]^−^, 329 [M+HCO_2_]^−^. ^1^H-NMR (400 MHz, CD_3_OD) *δ*: 7.44–7.26 (5H, m, Ph-H_5_ in PhCH_2_), 4.76 and 4.64 (each 1H, d, *J* = 11.9 Hz, PhCH_2_), 4.64 (1H, d, *J* = 3.6 Hz, H-1), 3.79 (1H, dd, *J* = 11.9, 2.3 Hz, H_a_-6), 3.74 (1H, t, *J* = 9.3 Hz, C-3), 3.65 (1H, dd, *J* = 11.9, 5.6 Hz, H_b_-6), 3.49 (1H, ddd, *J* = 9.8, 5.6, 2.3 Hz, H-5), 3.34 (3H, s, OCH_3_), 3.33–3.26 (2H, m, H-2,4). ^13^C-NMR (100 MHz, CD_3_OD) *δ*: 139.8, 129.4 (2C), 129.2 (2C), 128.9, all Ph-C_6_ in PhCH_2_; 99.2 (C-1), 81.0 (C-2), 74.3 (C-3), 74.0 (PhCH_2_), 73.3 (C-5), 71.8 (C-4), 62.6 (C-6), 55.4 (OCH_3_).

A mixed solution of **26** (2.47 g, 0.0087 mol) and **13** (9.5 g, 0.0207 mol) in anhydrous pyridine (50 mL) was stirred at 60 °C for 48 h and then evaporated under reduced pressure to give a reaction mixture that was separated by silica gel column chromatography (bed, 5.0 × 15.0 cm; eluted by a gradient of Pet. Et_2_O-EtOAc 6:1→4:1→2:1) to afford crude **27** (3.1 g, 0.0027 mol, 31.0% yield), eluted by Pet. Et_2_O-EtOAc (2:1), and **28** (5.5 g, 0.0049 mol, 56.3% yield), eluted by Pet. Et_2_O-EtOAc (4:1). The crude **28** (5.5 g, 0.0049 mol) was dissolved in THF-95% EtOH (10:2, 120 mL), transferred into a 0.5 L high-pressure reactor, and reduced by H_2_ under 10 atmospheric pressure at 40 °C for 12 h using 10% Pd–C (0.6 g) as catalyst. The reaction mixture was filtered to filter out the insoluble substances and evaporated under reduced pressure to give a reaction product that was separated by column chromatography on Sephadex LH-20 (bed, 2.8 × 60.0 cm; eluted by 50% EtOH) to afford pure *methyl 3,6-di-O-galloyl-α-d-glucopyranoside* (**9**, 0.5 g, 0.0010 mol) in the 20.4% yield. A crystalline powder (MeOH), m.p. 154–156 °C; ${\left[\alpha \right]}_{\text{D}}^{25}$ +109.0 (*c* 0.51, MeOH); positive ion ESI-MS *m/z*: 521 [M+Na]^+^, 537 [M+K]^+^; negative ion ESI-MS *m/z*: 497 [M−H]^−^. ^1^H- and ^13^C-NMR: Table 4 and Table 5. Similar reduction of crude **27** (3.1 g, 0.0027 mol), followed by separation of reduction product by Sephadex LH-20 column (bed, 2.8 × 60.0 cm; eluted by 40% EtOH) afforded additional amount of **8** (0.5 mg, 0.0010 mol) for a 37.0% yield.

#### *Methyl 3,6-Di-O-galloyl-α-d-glucopyranoside* (**10**)

A mixed solution of **26** (0.6 g, 0.0021 mol) and **13** (5.4 g, 0.0118 mol) in anhydrous pyridine (30 mL) was stirred at 60 °C for 48 h and then evaporated under reduced pressure to give a reaction mixture which was separated by silica gel column chromatography (bed, 6.0 × 8.0 cm; eluted by a gradient of Pet. Et_2_O-CH_2_Cl_2_ 100:0→0:100) to give crude **29** (2.5 g, 0.0016 mol, yield 76.2%). A portion of the crude **29** (2.3 g, 0.0015 mol) was dissolved in THF-95% EtOH (10:1, 110 mL), transferred into a 0.3 L high-pressure reactor, and reduced by H_2_ under 10 atmospheric pressure at 40 °C for 12 h using 10% Pd-C (0.4 g) as catalyst. The reaction mixture was filtered to filter out the insoluble substances and evaporated under reduced pressure to give a reduction product. This product was separated by Sephadex LH-20 column chromatography (bed, 2.0 × 70.0 cm; eluted by H_2_O-EtOH 4:6) to afford *methyl 3,6-di-O-galloyl-α-d-glucopyranoside* (**10**, 0.8 g, 0.0012 mol, 80.0% yield). A crystalline powder (MeOH), m.p. 171–173 °C; ${\left[\alpha \right]}_{\text{D}}^{25}$ +30.6 (*c* 0.49, MeOH); positive ion ESI-MS *m/z*: 651 [M+H]^+^, 668 [M+NH_4_]^+^, 673 [M+Na]^+^; negative ion ESI-MS *m/z*: 649 [M−H]^−^, 685 [M+Cl]^−^. ^1^H- and ^13^C-NMR: Table 4 and Table 5.

#### *Ethyl 2,3-Di-O-galloyl-α-d-glucopyranoside* (**11**) and *Ethyl 2,3-di-O-galloyl-β-d-glucopyranoside* (**12**)

A mixed solution of **17** (2.0 g, 0.0068 mol) and **13** (7.2 g, 0.0157 mol) in anhydrous pyridine (40 mL) was stirred at 60 °C for 48 h and then evaporated under reduced pressure to give a reaction mixture that was separated by silica gel column chromatography (bed, 3.8 × 13.0 cm; eluted by a gradient of Pet. Et_2_O-CH_2_Cl_2_ 2:1→CH_2_Cl_2_-MeOH 30:1) to afford crude **30** (a 1*α*,1*β* mixture, 7.3 g, 0.0064 mol, 94.1% yield). The crude 30 without further purification was dissolved in THF-95% EtOH (14:1, 150 mL), transferred into a 0.5 L high-pressure reactor, and reduced by H_2_ under 10 atmospheric pressure at 40 °C for 12 h using 10% Pd-C (1.5 g) as catalyst. The reaction mixture was filtered to filter out the insoluble substances and evaporated under reduced pressure to give a reduction product which was then separated by repeated Sephadex LH-20 column chromatography (bed, 2.8 × 60.0 cm; eluted by H_2_O-EtOH 7:3) to afford a mixture of crude **11** and **12** (total 1.4 g, 0.0027 mol, 42.2% yield). This mixture was further separated by preparative HPLC on a Capcell Pak C18 column (MG II S5, 2.0 × 25.0 cm), eluted by 30% MeOH, to obtain **11** (0.76 g, 0.0015 mol; *t*_R_ = 36.0 min) and **12** (0.55 g, 0.0011 mol; *t*_R_ = 23.0 min) in yields of 22.7% and 17.2%, respectively. *Ethyl 2,3-di-O-galloyl-α-d-glucopyranoside* (**11**): A crystalline powder (MeOH), m.p. 157–159 °C; ${\left[\text{α}\right]}_{\text{D}}^{25}$ +186.4 (*c* 0.44, MeOH); positive ion ESI-MS *m/z*: 513 [M+H]^+^, 535 [M+Na]^+^; negative ion ESI-MS *m/z*: 511 [M−H]^−^, 547 [M−Cl]^−^. UV λ_max_ nm (log *ε*) in MeOH: 223.5 (4.17), 276.4 (4.06). IR ν_max_ cm^−1^ (diamond ATR crystal): 3427, 2988, 2941, 1700, 1613, 1534, 1450, 1338, 1224. ^1^H- and ^13^C-NMR: Table 4 and Table 5. *Ethyl 2,3-di-O-galloyl-β-d-glucopyranoside* (**12**): A crystalline powder (MeOH), m.p. 160–162 °C; ${\left[\text{α}\right]}_{\text{D}}^{25}$ +98.6 (*c* 0.51, MeOH); positive ion ESI-MS *m/z*: 513 [M+H]^+^, 535 [M+Na]^+^; negative ion ESI-MS *m/z*: 511 [M−H]^−^, 547 [M+Cl]^−^. UV λ_max_ nm (log *ε*) in MeOH: 220.1 (4.14), 276.4 (3.91). IR ν_max_ cm^−1^ (diamond ATR crystal): 3400, 2986, 2948, 1704, 1613, 1532, 1381, 1325, 1241. ^1^H- and ^13^C-NMR: Table 4 and Table 5.

#### *Tri-O-benzylgalloyl chloride* (**13**)

To a solution of anhydrous K_2_CO_3_ (200 g) in DMSO (500 mL) was added dried gallic acid (60 g, 0.3529 mol) under stirring and heated to 140 °C. Then, benzyl chloride (300 mL) was added dropwise over 1.5 h under nitrogen atmosphere and then reacted at 140 °C for 8 h. After cooling down the reaction solution to the room temperature, the reaction mixture was diluted with cooled-distilled water (2000 mL) and extracted with CH_2_Cl_2_ (2000 mL × 4). The CH_2_Cl_2_ solution was combined and evaporated under reduced pressure to give a solid product. This product was suspended in MeOH (1800 mL), harvested by filtration, and washed with a suitable amount of methanol in the same filter to give a white powder. The powder was dried *in vacuo* and dissolved in CH_2_Cl_2_ (1000 mL). To the solution, MeOH (approximately 1000 mL) was added in portions, and when a lot of fine crystal species appeared, held at room temperature for two hours. Then, the crystals formed in the solution were filtered and then dried *in vacuo* to obtain *benzyl tri-O-benzylgallate* (135 g, 0.2547 mol, 72.1% yield). Fine needles (from CH_2_Cl_2_-MeOH), m.p. 91–93 °C; positive ESI-MS *m/z*: 548 [M+NH_4_]^+^. ^1^H-NMR (400 MHz, CDCl_3_) *δ*: 7.37–7.12 (22H, m, H-2,6 and Ph-H_5_ in PhCH_2_ × 4), 5.24 (2H, s, PhCH_2_), 5.03 (6H, s, PhCH_2_ ×3). ^13^C-NMR (100 MHz, CDCl_3_) *δ*: 166.1 (CO), 152.6 (2C, C-3,5), 142.5 (C-4); 137.5, 136.7 (2C), 136.1, 128.7–127.6 (20C), all Ph-C_6_ in PhCH_2_ × 4; 125.2 (C-1), 109.2 (2C, C-2,6); 75.2, 71.3 (2C), 66.9, all PhCH_2_ × 4.

To a solution of NaOH (200 g) in distilled water (500 mL) were added the benzyl tri-*O*-benzylgallate (135 g, 0.2547 mol) and MeOH (700 mL), in turn, and the mixture was refluxed at 90 °C for 5 h to hydrolyze the ester bond in the benzyl tri-*O*-benzylgallate by the base-catalyzed reaction. The solution was poured, after cooling to room temperature, into aqueous 1.2 N HCl (2500 mL) to neutralize the NaOH and stirred at room temperature for 30 min to precipitate the hydrolyzed product. The precipitate was filtered, washed with distilled water, and dried *in vacuo*. The precipitate was suspended in CH_2_Cl_2_-MeOH (3:1, 2000 mL) and refluxed to dissolve the precipitate in full, and the solution was kept at 4 °C overnight for crystallization. Then, the formed crystals in the solution were filtered, washed with MeOH, and dried *in vacuo* to obtain pure *tri-O-benzyl gallic acid* (100 g, 0.2273 mol, 89.2% yield). Fine needles (CH_2_Cl_2_-MeOH), m.p. 183–185 °C; positive ESI-MS *m/z*: 441 [M+H]^+^, 463 [M+Na]^+^. ^1^H-NMR (400 MHz, CDCl_3_) *δ*: 12.9 (1H, br s, COOH), 7.23–7.53 (17H, m, H-2,6 and Ph-H_5_ in PhCH_2_ × 3), 5.18 (4H, s, PhCH_2_ × 2), 5.05 (2H, s, PhCH_2_). ^13^C-NMR (100 MHz, CDCl_3_) *δ*: 166.8 (COOH), 152.0 (2C, C-3,5), 141.0 (C-4); 137.4, 136.8 (2C), 127.6–128.4 (15C), all Ph-C_6_ in PhCH_2_ × 3; 126.0 (C-1), 108.2 (2C, C-2,6); 74.2, 70.2 (2C), all PhCH_2_ × 3.

The tri-*O*-benzyl gallic acid (27 g, 0.0614 mol) was dissolved in SOCl_2_ (400 mL) and refluxed at 90 °C for 9 h. The solution was evaporated under reduced pressure to give a reaction mixture that was dried *in vacuo* and then dissolved in cyclohexane (600 mL) by refluxing. The solution was kept at room temperature overnight, and the crystals formed in the solution were collected by vacuum filtration, washed with cyclohexane, and dried *in vacuo* to obtain *tri-O-benzylgalloyl chloride* (**13**, 25 g, 0.0546 mol, 89.0% yield). Needles (from cyclohexane), m.p. 113–115 °C; positive ESI-MS *m/z*: 459 [M+H]^+^. ^1^H-NMR (400 MHz, CDCl_3_) *δ*: 7.22–7.56 (17H, m, H-2,6 and Ph-H_5_ in PhCH_2_ × 3), 5.18 (4H, s, PhCH_2_ × 2), 5.04 (2H, s, PhCH_2_). ^13^C-NMR (100 MHz, CDCl_3_) *δ*: 166.9 (CO), 152.0 (2C, C-3,5), 141.0 (C-4); 137.4, 136.8 (2C), 127.6–128.4 (15C), all Ph-C_6_ in PhCH_2_ × 3; 126.1 (C-1), 108.2 (2C, C-2,6); 74.3, 70.2 (2C), all PhCH_2_ × 3.

#### *Methyl 4,6-O-Benzylidene-α-d-glucopyranoside* (**14**)

To stirring MeOH (700 mL) acetyl chloride (32 mL) was added, followed in turn by dried d-glucose (100 g, 0.55 mol), and the solution was refluxed for 72 h and then evaporated under reduced pressure to give a reaction mixture that was separated by silica gel column chromatography (bed: 8.0 × 15.0 cm; eluted by CH_2_Cl_2_-MeOH 7:1), followed by further purification through recrystallization in EtOH to obtain pure *methyl α-d-glucopyranoside* (50 g, 0.26 mol, 47.3% yield). Needles (from EtOH), m.p. 163–165 °C; positive ESI-MS *m/z*: 212 [M+NH_4_]^+^, 217 [M+Na]^+^; negative ESI-MS *m/z*: 193 [M−H]^−^. ^1^H-NMR (400 MHz, CD_3_OD) *δ*: 4.67 (1H, d, *J* = 3.7 Hz, H-1), 4.61 (1H, t, *J* = 9.4 Hz, H-3), 3.81 (1H, dd, *J* = 11.8, 2.3 Hz, H_a_-6), 3.67 (1H, dd, *J* = 11.8, 5.7 Hz, H_b_-6), 3.52 (1H, ddd, *J* = 9.9, 5.7, 2.3 Hz, H-5), 3.39 (1H, dd, *J* = 9.4, 3.7 Hz, H-2), 3.41 (s, OCH_3_), 3.28 (1H, dd, *J* = 9.9, 9.4 Hz, H-4). ^13^C-NMR (100 MHz, CD_3_OD) *δ*: 101.2 (C-1), 75.1 (C-3), 73.5 (2C, C-2,5), 71.7 (C-4), 62.6 (C-6), 55.5 (OCH_3_).

A mixture of methyl-*α*-d-glucopyranoside (20 g, 0.10 mol), freshly distilled benzaldehyde (60 mL), triethyl orthoformate (20 mL), *p*-toluenesulfonic acid monohydrate (1.0 g) and THF (200 mL) was refluxed at 85 °C for 16 h. After cooling the reaction mixture to room temperature, K_2_CO_3_ (1.0 g) was added and the mixture was stirred at room temperature for 30 min. The mixture was filtered, and the filtrate was suspended in distilled water (400 mL) and a suitable amount of 95% EtOH was added to dissolve the suspended materials in full. Then, the solution was evaporated under reduced pressure, until a fine crystalline species appeared, and kept at 4 °C for 12 h. The crystals formed in the solution were filtered, washed with water (100 mL ×2) and cyclohexane (100 mL ×3), and dried *in vacuo* to give pure *methyl 4,6-O-benzylidene-α-d-glucopyranoside* (**14**, 20 g, 0.071 mol, 71.0% yield). Needles (EtOH-H_2_O), m.p. 163–165 °C; positive ion ESI-MS *m/z*: 283 [M+H]^+^, 300 [M+NH_4_]^+^, 305 [M+Na]^+^; negative ESI-MS *m/z*: 327 [M+HCO_2_]^−^. ^1^H-NMR (400 MHz, CDCl_3_) *δ*: 7.52–7.46 (2H, m, Ph-H_2_ in PhCH), 7.40–7.34 (3H, m, Ph-H_3_ in PhCH), 5.52 (1H, s, PhCH), 4.76 (1H, d, *J* = 3.9 Hz H-1), 4.28 (1H, dd, *J* = 9.8, 4.3 Hz, H_a_-6), 3.91 (1H, t, *J* = 9.3 Hz, H-3), 3.79 (1H, ddd, *J* = 9.8, 9.3, 4.3 Hz, H-5), 3.73 (1H, t, *J* = 9.8 Hz, H_b_-6), 3.60 (1H, dd, *J* = 9.3, 3.9 Hz, H-2), 3.47 (1H, t, *J* = 9.3 Hz, H-4), 3.44 (3H, s, OCH_3_). ^13^C-NMR (100 MHz, CDCl_3_) *δ*: 137.1, 129.4, 128.5 (2C), 126.4 (2C), all Ph-C_6_ in PhCH; 102.1 (PhCH), 99.9 (C-1), 81.0 (C-4), 72.9 (C-2), 71.8 (C-3), 69.0 (C-6), 62.4 (C-5), 55.7 (OCH_3_).

#### *Methyl 2,3-di-O-Benzyl-4,6-O-benzylidene-α-d-glucopyranoside* (**15**)

A mixed solution of **14** (17.5 g, 0.062 mol) and NaH (5 g) in DMF (300 mL) was stirred for 30 min, during which it became hot and was thus cooled down to room temperature. To the mixture benzyl bromide (45 mL) was added dropwise with stirring and reacted at room temperature for 4 h. To the reaction mixture was added distilled water (200 mL), and then it was extracted with CHCl_3_ (500 mL). The CHCl_3_ solution was washed with water (200 mL × 3), then dried with anhydrous MgSO_4_, filtered, and evaporated under reduced pressure, at a lower temperature at first to remove CHCl_3_ and further at 90 °C to remove the remaining DMF, to obtain a yellow-colored product. This product was separated by silica gel column chromatography (bed, 2.0 × 50.0 cm), eluted with Pet. Et_2_O–EtOAc (10:1), to obtain a fraction containing the target product **15**. Recrystallization of the fraction in Pet. Et_2_O gave **15** (25 g, 0.054 mol) in 87.1% yield. Fine needles (Pet.Et_2_O), m.p. 91–93 °C; positive ion ESI-MS *m/z*: 463 [M+H]^+^, 480 [M+NH_4_]^+^, 485 [M+Na]^+^. ^1^H-NMR (400 MHz, CDCl_3_) *δ*: 7.54–7.24 (15H, m, Ph-H_5_ in PhCH_2_ × 2 and PhCH × 1), 5.47 (1H, s, PhCH), 4.93 and 4.76 (each 1H, d, *J* = 11.2 Hz, PhCH_2_), 4.78 and 4.62 (each 1H, d, *J* = 12.4 Hz, PhCH_2_), 4.61 (1H, d, *J* = 3.6 Hz, H-1), 4.28 (1H, dd, *J* = 9.7, 4.7 Hz, H_a_-6), 4.07 (1H, t, *J* = 9.7 Hz, H-3), 3.85 (1H, td, *J* = 9.7, 4.7 Hz, H-5), 3.72 (1H, t, *J* = 9.7 Hz, H_b_-6), 3.62 (1H, t, *J* = 9.7 Hz, H-4), 3.57 (1H, dd, *J* = 9.7, 3.6 Hz, H-2), 3.42 (3H, s, OCH_3_). ^13^C-NMR (100 MHz, CDCl_3_) *δ*: 138.8, 138.2, 137.5, 129.0, 128.6 (2C), 128.4 (2C), 128.3 (2C), 128.2 (2C), 128.1 (2C), 128.0, 127.7, 126.1 (2C), all Ph-C_6_ in PhCH_2_ × 2 and PhCH × 1; 101.3 (PhCH), 99.3 (C-1), 82.2 (C-4), 79.2 (C-2), 78.7 (C-3), 75.5 (PhCH_2_), 73.9 (PhCH_2_), 69.2 (C-6), 62.4 (C-5), 55.5 (OCH_3_).

#### *4,6-O-Benzylidene-d-glucopyranose* (**16**)

A mixture of d-glucose (10 g, 0.0555 mol), newly distilled benzaldehyde (20 mL), *p*-toluenesulfonic acid monohydrate (1.0 g) and DMF (100 mL) was refluxed at 45 °C for 16 h. After the reaction mixture was cooled to room temperature, K_2_CO_3_ (1.0 g) was added into the mixture, stirred for 30 min, and filtrated to obtain a filtrate. To the filtrate was added distilled water (300 mL), and then the mixture was concentrated under reduced pressure to give a slimy product, which was separated by silica gel column chromatography (bed, 3.5 × 10.0 cm), eluted by a gradient of CH_2_Cl_2_→CH_2_Cl_2_-MeOH (15:1), to afford **16** (9.5g, 0.0354 mol, yield 63.8%). A crystalline powder (CH_2_Cl_2_-MeOH), m.p. 161–163 °C; positive ion ESI-MS *m/z*: 269 [M+H]^+^, 291 [M+Na]^+^, 559 [2M+Na]^+^; negative ion ESI-MS *m/z*: 267 [M−H]^−^, 303 [M+Cl]^−^, 313 [M+HCO_2_]^−^. ^1^H-NMR (400 MHz, CD_3_COCD_3_) *δ* for *α*-isomer: 7.52–7.46 (2H, m, Ph-H_2_ in PhCH), 7.40–7.32 (3H, m, Ph-H_3_ in PhCH), 5.58 (1H, s, PhCH), 5.18 (1H, d, *J* = 3.8 Hz, H-1), 4.14 (1H, dd, *J* = 10.1, 4.9 Hz, H_a_-6), 3.96 (1H, td, *J* = 9.2, 4.9 Hz, H-5), 3.87 (1H, t, *J* = 9.2 Hz, H-3), 3.70 (1H, dd, *J* = 10.1, 9.2 Hz, H_b_-6), 3.48 (1H, dd, *J* = 9.2, 3.8 Hz, H-2), 3.43 (1H, t, *J* = 9.2 Hz, H-4); *δ* for *β*-isomer: 7.52–7.46 (2H, m, Ph-H_2_ in PhCH), 7.40–7.32 (3H, m, Ph-H_3_ in PhCH), 5.58 (1H, s, PhCH), 4.65 (1H, d, *J* = 7.7 Hz, H-1), 4.20 (1H, dd, *J* = 10.2, 4.6 Hz, H_a_-6), 3.72 (1H, dd, *J* = 10.2, 8.8 Hz, H_b_-6), 3.46 (1H, t, *J* = 8.8 Hz, H-4), 3.41 (1H, td, *J* = 8.8, 4.6 Hz, H-5), 3.28 (1H, dd, *J* = 8.8, 7.7 Hz, H-2). ^13^C-NMR (100 MHz, CD_3_COCD_3_) *δ* for *α*-isomer: 139.3, 129.4 (2C), 128.7, 127.3 (2C), all Ph-C_6_ in PhCH; 102.2 (PhCH), 94.3 (C-1), 82.8 (C-4), 74.3 (C-2), 71.7 (C-3), 69.7 (C-6), 63.2 (C-5); *δ* for *β*-isomer: 139.2, 129.4 (2C), 128.7, 127.3 (2C), all Ph-C_6_ in PhCH; 102.0 (PhCH), 98.8 (C-1), 82.1 (C-4), 77.2 (C-2), 74.4 (C-3), 69.3 (C-6), 67.2 (C-5).

#### *Ethyl 4,6-O-Benzylidene-d-glucopyranoside* (**17**)

A mixed solution of d-glucose (20 g, 0.1111 mol), newly distilled benzaldehyde (45 mL), triethyl orthoformate (20 mL), *p*-toluenesulfonic acid monohydrate (1.0 g) and THF (200 mL) was refluxed at 85 °C for 16 h. After the reaction mixture cooled down to room temperature, K_2_CO_3_ (1.0 g) was added to the mixture, which was stirred for 30 min, and filtered to obtain a filtrate. The filtrate was concentrated under reduced pressure and then separated by column chromatography on silica gel (bed, 5.0 × 15.0 cm), using a gradient of CH_2_Cl_2_→CH_2_Cl_2_-EtOH (20:1) as eluting solvent, to afford **17** (10 g, 0.0338 mol, 30.4% yield) as a mixture of *α*,*β*-isomers. An amorphous powder (CH_2_Cl_2_-MeOH), positive ion ESI-MS *m/z*: 297 [M+H]^+^, 314 [M+NH_4_]^+^, 319 [M+Na]^+^; negative ion ESI-MS *m/z*: 341 [M+HCO_2_]^−^. A portion of **17** (approximate 250 mg) was separated by preparative HPLC (Capcell Pak C18, MG II S5, 20 × 250 mm, 50% MeOH, 10 mL/min) to obtain *α*-isomer **17a** (105 mg, *t*_R_ = 50.7 min) and *β*-isomer **17b** (100 mg, *t*_R_ = 36.3 min). *Ethyl 4,6-O-benzylidene-α-d-glucopyranoside* (**17a**): positive ion ESI-MS *m/z*: 297 [M+H]^+^, 314 [M+NH_4_]^+^; negative ion ESI-MS *m/z*: 341 [M+HCO_2_]^−^. ^1^H-NMR (400 MHz, CD_3_OD) *δ*: 7.53–7.46 (2H, m, Ph-H_2_ in PhCH), 7.39–7.31 (3H, m, Ph-H_3_ in PhCH), 5.57 (1H, s, PhCH), 4.84 (1H, d, *J* = 4.0 Hz, H-1), 4.20 (1H, dd, *J* = 8.9, 3.5 Hz, H_a_-6), 3.88–3.70 (3H, m, H_b_-6, H-5 and Ha in OCH_2_CH_3_), 3.84 (1H, t, *J* = 9.3 Hz, H-3), 3.64–3.41 (1H, m, Hb in OCH_2_CH_3_), 3.51 (1H, dd, *J* = 9.3, 4.0 Hz, H-2), 3.45 (1H, t, *J* = 9.3 Hz, H-4), 1.27 (3H, t, *J* = 7.1 Hz, OCH_2_CH_3_). ^13^C-NMR (100 MHz, CD_3_OD) *δ*: 129.9, 129.0 (2C), 127.5 (2C), 139.2, Ph-C_6_ in PhCH; 103.3 (PhCH), 100.7 (C-1), 83.0 (C-4), 74.0 (C-2), 72.0 (C-3), 70.0 (C-6), 64.9 (OCH_2_CH_3_), 64.0 (C-5), 15.4 (OCH_2_CH_3_). *Ethyl 4,6-O-benzylidene-β-d-glucopyranoside* (**17b**): positive ion ESI-MS *m/z*: 297 [M+H]^+^, 314 [M+NH_4_]^+^, 615 [2M+Na]^+^. ^1^H-NMR (400 MHz, CD_3_OD) *δ*: 7.54–7.47 (2H, m, Ph-H_2_ in PhCH), 7.39–7.31 (3H, m, Ph-H_3_ in PhCH), 5.57 (1H, s, CHPh), 4.40 (1H, d, *J* =7.8 Hz, H-1), 4.28 (1H, dd, *J* = 10.1, 4.4 Hz, H_a_-6), 3.91 (1H, dq, *J* = 9.4, 7.1 Hz, Ha in OCH_2_CH_3_), 3.76 (1H, t, *J* = 10.1 Hz, H_b_-6), 3.68–3.59 (2H, m, H-3 and Hb in OCH_2_CH_3_), 3.50–3.39 (1H, m, H-5), 3.48 (1H, t, *J* = 9.2 Hz, H-4), 3.27 (1H, dd, *J* = 9.1, 7.8 Hz, H-2), 1.24 (3H, t, *J* = 7.1 Hz, OCH_2_CH_3_). ^13^C-NMR (100 MHz, CD_3_OD) *δ*: 129.9, 129.0 (2C), 127.5 (2C), 139.1, Ph-C_6_ in PhCH; 104.8 (C-1), 102.9 (PhCH), 82.3 (C-4), 75.9 (C-2), 74.6 (C-3), 69.7 (C-6), 67.6 (C-5), 66.6 (OCH_2_CH_3_), 15.5 (OCH_2_CH_3_).

### 3.3. MTT Assay

Compounds **1–12**, 5-FU, DOC and taxol were dissolved in DMSO to prepare 10.0 mg/mL stock solutions, respectively, and serial dilutions were made for the MTT assay. 5-FU, DOC and taxol were used as positive control, and DMSO was used as blank control. The MTT assay was performed according to our previous procedure [30,31,32,33,34,35,36,37,38,39,40,41,42,43,44], and exponentially growing K562, HL-60 and HeLa cells in RPMI 1640 medium and S180 cells in DMEM medium were treated with samples at 37 °C for 48 h. The assay was run in triplicate, and the OD value was read at 570 nm. The IR% was calculated using OD mean values by the formula, IR% = (OD_control_ − OD_sample_)/OD_control_ × 100%, and the IC_50_ value for a sample was obtained from its IR% values at different concentrations.

### 3.4. The in Vivo Test in Mice for Antitumor Activity of ***1*** and ***2***

Compounds **1** and **2** (30 mg of each) and taxol (20 mg) was dissolved in DMSO (1 mL) and mixed with oxidized castor oil (1 mL) to prepare stock solutions of **1** and **2** at 15 mg/mL and taxol at 10 mg/mL, respectively. Stock solutions were repeatedly prepared according to the need. Before administration, each 0.4 mL of the stock solution was diluted with 3.6 mL of 5% glucose aqueous solution to obtain diluted solutions of **1** and **2** at 1.5 mg/mL and taxol at 1.0 mg/mL for each round of administrations. A blank solvent without test samples was also prepared in the same manner and used for the model group as blank control.

The *in vivo* antitumor activity of **1** and **2** was tested by a procedure similar to that described in [52,53]. Sixty three Kunming mice (18–20), which had received a hypodermic injection of murine sarcoma S180 cells (0.2 mL each of fresh cell suspensions at density of 1 × 10^7^ cells/mL) into the armpit, were randomly divided into six groups: the model (blank solvent) group with 11 mice, positive control taxol (20 mg/kg) group with eight mice, and four test groups with 11 mice in each group for **1** and **2** at both 15 and 30 mg/kg, respectively. The drug administration was performed by intravenous injection via the mouse tail vein from the next day of tumor cell injection. The four test groups were injected every day continuously 6 days, while the taxol group was injected every other day for 6 days (for a total of three administrations). The model group was continuously injected the same volume of blank solvent as that of 30 mg/kg test sample solutions every day for 6 days. On the third day of the last administration, the mice were sacrificed, the body and tumor weights were weighed, and the inhibition rate of **1–2** and taxol on the tumor growth was calculated as described in the main text. Statistical analysis for the test and positive control taxol groups was performed by the Student’s *t* test using body and tumor weights in mean ± S.D. values in comparison with the model group.

## 4. Conclusions

Twelve galloyl glucosides **1–12** with two or three galloyl groups were synthesized from d-glucose and gallic acid. Three of them, **9**, **11** and **12**, were new compounds and six others, **1–4**, **6** and **10**, were synthesized for the first time. In an *in vitro* MTT assay, **1–12** inhibited human cancer K562, HL-60 and HeLa cells with inhibition rates ranging from 64.2% to 92.9% at 100 μg/mL and their IC_50_ values were determined to vary between 17.2–124.7 μM. In addition, **1–12** also inhibited murine sarcoma S180 cells with inhibition rates ranging from 38.7% to 52.8% at 100 μg/mL in an *in vitro* MTT assay, and the *in vivo* antitumor activity of **1** and **2** was also detected on murine sarcoma S180 tumor-bearing Kunming mice using taxol as positive control.
